# A national cross-sectional survey and interviews exploring the relationship between well-being and burnout in doctors

**DOI:** 10.1192/bjo.2021.139

**Published:** 2021-06-18

**Authors:** John Jenkins, Emma Boxley, Gemma Simons

**Affiliations:** 1 University of Southampton; 2 Centre for Workforce Wellbeing, University of southampton

## Abstract

**Aims:**

Doctors’ mental health is a national concern – the General Medical Council, British Medical Association and Health Education England pledge to improve their well-being. Well-being has no common definition, instead pathogenic measures such as burnout are published as a demonstration of doctors’ wellbeing. Yet, the relationship between burnout and wel-being has not been explored.

**Aim:**

to investigate the relationship between burnout and well-being.

**Hypothesis:**

they are negatively associated, but not opposites.

**Method:**

An online cross-sectional national survey was distributed to doctors of all grades and specialties via the Royal Colleges and doctor organisations. The Oldenburg Burnout Inventory (OLBI) measured burnout, and the Warwick-Edinburgh Mental Wellbeing Scale (WEMWBS) measured well-being. Correlation coefficients between total scores of these measures estimated the relationship. Additionally, semi-structured interviews explored personal definitions of wellbeing and its relationship with burnout. Thematic analysis was carried out.

**Result:**

64 doctors completed the OLBI and WEMWBS. Comparing the total scores for the questionnaires with Spearman's rho indicates a moderate negative correlation (rs= –0.658, p = 0.00, n = 64). Total scores were made into binary variables, a Chi-square test showed that a low WEMWBS score (<40) and a very high risk OLBI score (≥2.85 exhaustion and ≥2.6 disengagement) were statistically significantly associated (X 2 (1, N = 64) = 4.232, p = 0.04). Three themes emerged from the 10 interviews conducted: the importance of networks/relationships outside work; scepticism towards the proposal of an NHS wellbeing check-in; and how participants do not strive to improve their wellbeing until its decline.

**Conclusion:**

This research demonstrates that wellbeing and burnout have only a moderate negative correlation when using commonly employed measurement tools. Therefore, measures of burnout are not a surrogate for wellbeing. Further research could adopt a salutogenic approach by using the WEMWBS to monitor doctors’ wellbeing and could explore interventions to increase well-being, rather than waiting for its decline.

